# NF-κB and tPA Signaling in Kidney and Other Diseases

**DOI:** 10.3390/cells9061348

**Published:** 2020-05-29

**Authors:** Samantha White, Ling Lin, Kebin Hu

**Affiliations:** Nephrology Research Program, Department of Medicine, Department of Cellular and Molecular Physiology, The Pennsylvania State University College of Medicine, Hershey, PA 17033, USA; swhite11@pennstatehealth.psu.edu

**Keywords:** nuclear factor-κB (NF-κB), tissue plasminogen activator (tPA), inflammation, macrophages, kidney fibrosis, kidney disease, ischemic stroke, cardiovascular disease

## Abstract

The activation of the nuclear factor-κB (NF-κB) pathway plays a central role in the initiation and progression of inflammation, which contributes to the pathogenesis and progression of various human diseases including kidney, brain, and other diseases. Tissue plasminogen activator (tPA), a serine protease regulating homeostasis of blood coagulation, fibrinolysis, and matrix degradation, has been shown to act as a cytokine to trigger profound receptor-mediated intracellular events, modulate the NF-κB pathway, and mediate organ dysfunction and injury. In this review, we focus on the current understanding of NF-κB and tPA signaling in the development and progression of kidney disease. Their roles in the nervous and cardiovascular system are also briefly discussed.

## 1. Introduction

Chronic kidney disease (CKD) is one of the most common chronic diseases in the world. CKD is also an important multiplier of risk for many chronic diseases [1], including cardiovascular disease [2] and cancer [3]. High blood pressure and diabetes are the most common initial causes of CKD. Patients with CKD are often undetected until they have entered later stages of the disease due to the fact that patients of early stages have few symptoms. Even worse, early kidney disease, if untreated, will progress into more advanced stages and ultimately into end-stage renal disease (ESRD), the stage of the disease with limited treatment options beyond dialysis or organ transplant. There are around 30 million people in the United States affected by CKD, as of 2019 [4]. It is unlikely that this high morbidity and associated cost will be reduced until we have a better understanding of the cellular and molecular mechanisms of CKD and develop a specific and effective therapy.

Regardless of the etiology, the onset of inflammation and the accumulation of fibrosis are the key characteristics of CKD. In response to a sustained injury, inflammatory signaling systems are activated in renal cells, leading to the production of pro-inflammatory chemokines and cytokines, recruiting immune cells such as monocytes, neutrophils, and macrophages to the damage site [5]. The inflammation is further exasperated as these cells stimulate the production of more pro-inflammatory genes by activating transcription factors, of which nuclear factor-κB (NF-κB) is the most predominant and well-studied one. NF-κB is a family of inducible transcription factors responsible for regulating the induction and advancement of inflammatory responses. Increased NF-κB activity is predominant in many diseases, including CKD [6]. Therefore, understanding the regulatory mechanisms of NF-κB holds potential for CKD treatments.

Tissue plasminogen activator (tPA) is a serine protease family member with dual functions as a protease and a cytokine. tPA is classically known for regulating fibrinolysis and matrix regulation due to its protease activities. However, recently, tPA has been shown to operate as a cytokine to regulate an array of intracellular signaling events [7]. Many studies have demonstrated that tPA expression, much like NF-κB, is also increased with the initiation and progression of CKD [8]. As discussed below, tPA regulates inflammatory responses as a cytokine by modulating the NF-κB pathway.

In this review, we will highlight the roles of tPA signaling and the NF-κB pathway in kidney and other diseases, such as cerebral ischemic stroke and cardiovascular diseases.

## 2. NF-κB Pathway

### 2.1. Discovery and Structure

NF-κB is a key family of inducible transcription factors responsible for regulating an array of immune and inflammatory responses. NF-κB was initially identified in 1986 as a DNA binding element in immunoglobulin kappa light chain in B lymphocytes, as its name reflects [9]. Today, it is known that the NF-κB family consists of five members: p50/NF-κB1, p52/NF-κB2, p65/RelA, RelB, and c-Rel, which can form a variety of homo- and hetero-dimers combinations. The most common heterodimer is p50/p65, which is detectable in nearly all cell types [10]. A common conserved feature among NF-κB family members revealed by structural studies is the Rel homology domain (RHD). RHD is a 300 amino acid long region that functions as the NF-κB site for homo-/hetero-dimerization, nuclear translocation, and DNA promoter binding [10,11]. A structural feature that subdivides the NF-κB family is based on their transactivation potential. Only p65/RelA, RelB, and c-Rel contain the carboxy-terminal transactivation domain (TAD) required to be transcriptionally active [10].

### 2.2. NF-κB Activation and Signaling Pathways

In an inactive state, NF-κB is sequestered in the cytoplasm by specific members of the inhibitory κB (IκB) family. The IκB proteins possess ankyrin repeat motifs which are able to interact with RHD of NF-κB members, effectively inhibiting and sequestering NF-κB [10]. Additionally, p105 and p100, the precursor molecules of p50/NF-κB1 and p52/NF-κB2, can inhibit some NF-κB members. P105 and p100 contain a C terminal region akin to IκB that possess ankyrin repeats, allowing them to similarly sequester NF-κB in the cytoplasm [12].

In order to function as a transcription factor, it is necessary for NF-κB to be activated in a variety of dimers formed through the combination of its members, translocate to the nucleus, and interact with DNA. NF-κB activation occurs via two main pathways: canonical (classic) and non-canonical (alternative). The canonical pathway is initiated when a variety of signals, such as proinflammatory cytokines and pathogen-associated molecular patterns (PAMPs), activate cell surface receptors, including pattern-recognition receptors (PRRs), toll-like receptors (TLR), and T-cell receptors (TCR). This triggers the activation of the IκB kinase (IKK), which is comprised of two catalytic subunits, IKKα and IKKβ, and one regulatory subunit, NF-κB essential modulator (NEMO)/IKKγ. Activated IKK then phosphorylates IκB proteins and p105, causing their ubiquitination and degradation by the proteasome. Without inhibition by IκB or p105, NF-κB members in their various dimers can be rapidly translocated into the nucleus. The NF-κB canonical signaling members include p50/NF-κB1, p65/RelA, and c-Rel, with the most common dimers being p50/NF-κB1-p65/RelA and p50/NF-κB1-c-Rel [13].

Compared to the broader applications of the canonical pathway, the non-canonical/alternative pathway responds to a more specific set of stimuli. Primarily, non-canonical signaling is activated by tumor necrosis factor (TNF) cytokines and their respective TNF receptors (TNFR). Although there are additional receptors that mediate the non-canonical NF-κB pathway, the TNFRs are the most well-known. Involved TNFRs include lymphotoxin-β receptor (LTβR), B cell activating factor receptor (BAFFR), fibroblast growth factor-inducible factor 14 (Fn14), and more [13]. Following TNF or other pertinent receptor stimulation, NF-κB inducing kinase (NIK) is activated to initiate the non-canonical pathway. A signaling cascade then proceeds, with NIK first phosphorylating IKKα, one of the catalytic subunits of IKK. Then IKKα subsequently phosphorylates p100, causing its IκB-like C terminal to be degraded [14]. As a result, p52/NF-κB2 is generated and the non-canonical dimer complex p52/NF-κB2-RelB will translocate to the nucleus.

### 2.3. NF-κB as a Regulator of Inflammation

Despite being originally identified in B cells, NF-κB is expressed in nearly all cell types and is now known to be a much more complex, the master regulator of inflammation. As a transcription factor, NF-κB is very important because it has the ability to induce the transcription of many pro-inflammatory genes, as summarized in various reviews regarding its activation and downstream targets [13,15]. It is important to point out that not only key inflammatory molecules, such as cytokines and chemokines, but also cellular stress inducers, such as ultraviolet (UV) irradiation and oxidative stress, can activate NF-κB signaling [16]. In terms of downstream targets, the action of the NF-κB family members is varied depending on the context and the cell types. Examples of NF-κB downstream targets are cytokines, cell cycle regulators, and growth factors [17]. Given its extensive role in inflammation responses, it is no surprise that the NF-κB pathway plays a central role in inflammatory diseases, such as rheumatoid arthritis, multiple sclerosis, inflammatory bowel disease (IBD), and atherosclerosis [18].

## 3. tPA Signaling

### 3.1. Discovery and Structure

tPA is a member of the serine protease superfamily. tPA was first identified in the year 1902 under the name of fibrikinase [19]. It was later characterized and purified in human blood vessels and the uterus in 1979 [20,21]. tPA expression is documented in many tissue types including the endothelial cells lining blood vessels, the kidneys, and the brain. Structurally, tPA is a 527 amino acid, 69 kDa glycoprotein [22]. tPA is first secreted as a single chain, and is subsequently cleaved into a two-chain active form with a heavy and a light chain. tPA has a total of five domains, with the heavy chain containing the first four domains and the light chain containing the final one. From the N to C terminus, the five domains are: finger (F) domain, Epidermal growth factor (EGF) domain, two Kringle (K1 and K2) domains, and serine protease catalytic (SPC) domain [22].

Each domain of tPA plays important functional roles. The F domain is necessary for tPA binding of fibrin and interactions with the tPA associated receptors, such as low-density lipoprotein receptor-related protein (LRP-1) and annexin A2. The EGF domain allows tPA to interact with EGF receptors. The Kringle domains contain active sites with an affinity for lysine which are thought to mediate binding and protein-protein interaction. Finally, the serine protease catalytic domain (also called the light chain) consists of a catalytic triad of Ser^478^, His^322^, and Asp^371^, which is involved in the conversion of plasminogen to plasmin [19,23].

### 3.2. Dual Function as a Protease and a Cytokine

tPA is most well-known as a plasminogen activator in the circulatory system and as a key mediator in blood clot degradation. In brief, as thrombosis or blood clot formation occurs, fibrinogen is converted into fibrin. Insoluble fibrin fibers are then formed into a mesh-like network ultimately impeding blood flow [24]. To break-down and dissolve the blood clot, a process calls fibrinolysis occurs. During fibrinolysis, the two known plasminogen activators, tPA and its cousin serine protease urokinase plasminogen activator (uPA), cleave plasminogen into plasmin [25,26]. Plasmin is an important active enzyme which degrades insoluble fibrin fibers into small fragments which can be processed and removed by other proteases and the kidneys. Therefore, tPA-mediated fibrinolysis is crucial for blood flow restoring in the diseases involving blood clots. In fact, tPA is currently the only approved U.S. Food and Drug Administration (FDA) treatment for ischemic stroke, which is caused by a cerebral artery blockage [27]. Furthermore, tPA can also regulate the degradation of extracellular matrix (ECM) through matrix metalloproteinases (MMPs), which are activated by plasmin [8].

Recently, studies from us and other groups have shown that tPA also functions as a cytokine [7]. tPA has been shown to promote the fibrosis through inducing matrix metalloproteinase-9 (MMP-9) expression in the injured kidney [28]. We further found that tPA-induced MMP-9 expression does not depend on its protease activity, but instead, through a signaling cascade with LRP-1 acting as a cell membrane receptor for tPA. It was noted that the tPA signaling mirrors hepatocyte growth factor (HGF), a well-known cytokine. Interestingly, both HGF and tPA contain similar Kringle domains, further prove that tPA can function as a cytokine [29,30]. This study and others have laid the foundation for the future illustration of tPA as a cytokine in various intracellular signaling events. Later on, tPA, as a cytokine, has now been implicated in the pathogenesis of numerous disease models including liver fibrosis, ischemic brain injury, and chronic kidney disease [8,31,32].

### 3.3. tPA Associated Receptors 

tPA does not have a specifically designated cell surface receptor. However, tPA is known to associate with the receptors LRP-1 and annexin A2. LRP-1 is a 600 kDa transmembrane protein first identified as a tPA receptor in hepatocytes [33]. LRP-1 contains two subunits α and β. The LRP-1 α subunit consists of an extracellular segment, while the β subunit contains the transmembrane and cytosolic tail portions [34]. tPA has been shown to bind domains II and IV in the α subunit and can induce the phosphorylation of a tyrosine site in the β subunit [35,36,37]. tPA and LRP-1 signaling has been noted in many organ systems including the kidneys, central nervous system, skin, and liver, with implications in conditions such as kidney fibrosis, melanoma, and ischemic brain diseases [38,39,40]. The interaction between tPA and LRP-1 mediates multiple signaling cascades and influences cellular processes including ECM remodeling, myofibroblast activation, fibroblast accumulation and proliferation [7,37,41].

Another recognized but less known receptor for tPA is annexin A2, a member of the calcium-independent phospholipid-binding protein family. Annexin A2 was first discovered as a tPA receptor in microglia of the central nervous system (CNS) [42]. It is of note that annexin A2 docks onto the plasma membrane in a peripheral fashion because it lacks a transmembrane domain. Therefore, it is thought that annexin A2 may possess additional co-receptors [43]. Past studies have demonstrated tPA/annexin A2 binding on endothelial and some cancer cells [44,45,46]. Structurally, annexin A2 is a 36 kDa protein containing three regions: the core region, the C terminal, and the N terminal. All annexin family members share a conserved core region of approximately 70 amino acids, also called the annexin repeat, which mediates the ligand binding. The N terminal region is varied among annexin family members, thus giving each annexin member a unique ability to interact with their ligands [47]. For annexin A2, tPA has been shown to bind to the N terminal region at residues 7 through 12 via a hexapeptide LCKLSL [48]. tPA and annexin A2 binding has been shown to play a role in reconstituting extracellular matrix, promoting cell migration, and activating microglia [42,46,49].

## 4. tPA and NF-κB in Kidney Disease

Key hallmarks of CKD include the presence of widespread inflammation and the accumulation of fibrosis. Following initial tissue injury, inflammation and the infiltration of innate immune cells such as lymphocytes, monocytes, and macrophages are thought to first ‘prime’ the organ and establish a profibrogenic environment, thus facilitating the onset of fibrosis [50]. In fact, the interconnection between the degree of inflammation and tubulointerstitial fibrosis has previously been demonstrated [50,51,52]. In terms of current research on chronic kidney disease, macrophages are one of the major inflammatory cells of interest because their accumulation is a common pathological feature in progressive kidney diseases. They contribute to chronic inflammation and fibrosis by excreting molecules that stimulate proinflammatory cytokines, growth factors, and reactive oxygen species (ROS), causing a cascade in which fibroblasts and ECM-producing cells are activated. With chronic inflammation, fibrosis is initiated as over-production and accumulation of ECM to form scar tissue [50]. Excessive fibrosis is consequential to the pathogenesis of kidney disease because it is an irreversible process, leading to loss of kidney function and ultimately organ failure, an obviously detrimental outcome for patients. The correlation between the degree of inflammation and kidney function has been shown in multiple human CKD studies [53,54].

### 4.1. NF-κB and Renal Inflammation

Both NF-κB and tPA has been implicated as mediators during renal inflammation and fibrosis. NF-κB has been shown to be associated with immune-related kidney diseases, including Lupus Nephritis and IgA nephropathy [55,56]. NF-κB activation also has been documented in animal models of kidney injury, including unilateral ureteral obstruction (UUO) and ischemia/reperfusion (I/R) [6,57,58]. In these studies on CKD animal models, NF-κB activation has been linked to increased tubulointerstitial fibrosis [59]. NF-κB activity has additionally been linked to acute kidney injury (AKI), which is clinically relevant because AKI often contributes to the onset of more severe CKD. Studies on NF-κB and AKI report that NF-κB inhibitors reduce AKI severity, even following the start of injury [60,61]. NF-κB inhibition has also been shown to reduce inflammatory responses and fibrosis in various CKD models, further confirming the importance of NF-κB as a renal inflammation mediator [62,63].

**Figure 1:**
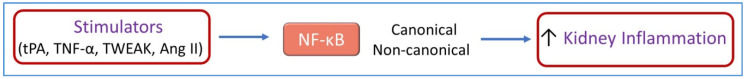
Nuclear factor-κB (NF-κB) activation in kidney disease. Various stimulators, such as Tissue plasminogen activator (tPA), tumor necrosis factor-α (TNF-α), TNF-like weak inducer of apoptosis (TWEAK), and angiotension II (Ang II), activate NF-κB through either the canonical or non-canonical pathway to promote renal inflammation.

It is important to better understand the underlying mechanisms which initiate and regulate NF-κB pathways in kidney diseases. As NF-κB can be activated by a variety of stimuli through either canonical or non-canonical signaling pathways, there are many possible activation triggers. The activation triggers that have been identified are varied depending on the underlying causes and types of kidney diseases. One such activator is TNF-α, which has been shown to stimulate the canonical NF-κB pathway in ischemia/reperfusion kidney injury [64]. Another activator of the both canonical and non-canonical NF-κB pathways in relation to kidney diseases is TNF-like weak inducer of apoptosis (TWEAK) and its associated receptor Fn14. TWEAK is a cytokine family that activates NF-κB and stimulate the expression of NF-κB-dependent pro-inflammatory chemokines like monocyte chemoattractant protein (MCP)-1 through the canonical pathway or chemokine (C-C motif) ligand 21 (CCL21) through the noncanonical pathway [65]. Angiotensin II, a hormone best known to regulate blood pressure, has also been shown to activate the NF-κB pathway to induce renal inflammation in UUO model [66,67] (Figure 1).

### 4.2. tPA Signaling in Kidney Fibrosis and Inflammation

**Figure 2:**
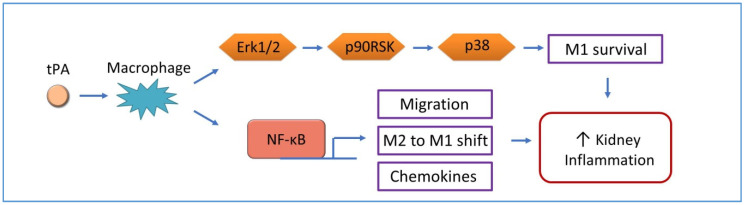
tPA signaling in kidney disease. tPA promotes kidney inflammation through NF-κB-dependent or independent pathways.

Recently, tPA has been implicated as an NF-κB activator in the pathogenesis of kidney disease. tPA is a prime candidate for NF-κB activation because of its concurrent induction with NF-κB during the progression of CKD, as well as its potent ability to modulate renal inflammatory responses [8,34]. Mounting evidence from various animal models of kidney injury and disease have shown that tPA deficiency has alleviated inflammatory responses and inflammatory infiltration, supporting the idea that tPA is a key player in promoting renal inflammation [31,68]. Moreover, tPA has been shown to directly protect key inflammatory cells [8]. A specific example is that tPA protects M1 macrophages from apoptosis through an novel intracellular signaling cascade involving extracellular signal-regulated kinase (ERK), the 90kDa ribosomal s6 kinase (p90RSK), and p38 [69] (Figure 2).

### 4.3. tPA Modulates Renal Inflammation through NF-κB in Macrophages

**Figure 3:**
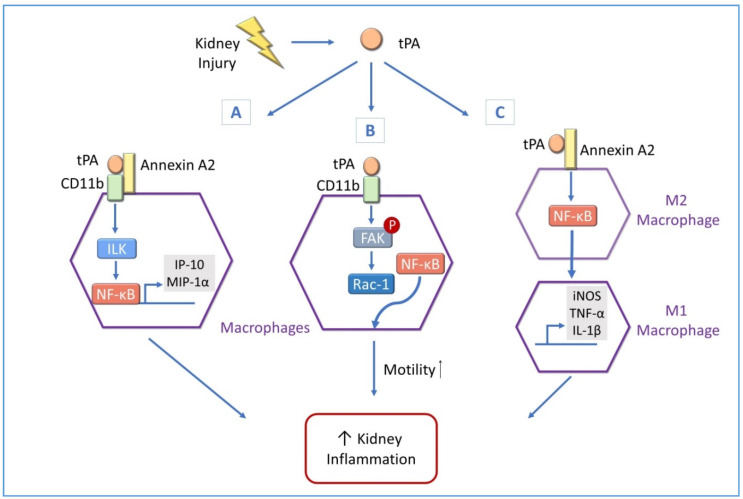
tPA-induced NF-κB activation in kidney disease. Following kidney injury, there is an increased level of tPA, which in turn mediates NF-κB activation in macrophages, leading to increased renal inflammation through three signal pathways: (**A**) tPA binds to annexin A2 and promotes aggregation of annexin A2 and CD11b leading to activation of the integrin-linked kinase (ILK)/NF-κB pathway and expression of NF-κB-dependent IP-10 and MIP-1 α. (**B**) tPA activates CD11b-dependent focal adhesion kinase (FAK) and the ras-related C3 botulinum toxin substrate 1 (Rac1) pathway. This novel signal cascade, together with tPA-induced NF-κB signaling, results in increased macrophage motility and ultimately kidney inflammation. (**C**) tPA promotes macrophage M2 to M1 phenotypic change through an annexin A2 and NF-κB-mediated pathway. Inducible nitric oxide (iNOS), TNF-α, and IL-1β are typical M1 chemokines.

Given the important role of NF-κB and tPA in kidney inflammation, we have proposed that tPA may mediate renal inflammation through NF-κB activation. First evidence supporting the idea has come from the research on macrophages. As previously mentioned, macrophage accumulation is a hallmark of CKD and therefore the role of macrophages during CKD has been of great interest. In human patients, there is a correlation between kidney disease severity and macrophage infiltration, suggesting these cells contribute to disease pathogenesis [70,71]. We have found that tPA induces the expression of the proinflammatory chemokines such as interferon-γ-inducible protein (IP)-10 and macrophage inflammatory protein (MIP)-1 α in macrophages and promote the infiltration of macrophages in an obstruction-induced CKD model. We further have demonstrated that tPA activates NF-κB signaling via the canonical pathway in macrophages by promoting IκB phosphorylation and stimulating translocation of p65/RelA to the nucleus [72], and annexin A2 mediates tPA-induced NF-κB activation. However, it is known that annexin A2 lacks a transmembrane domain; the underline signaling mechanism remains to be elucidated. We have hypothesized that annexin A2 may act through a co-receptor. Co-immunoprecipitation studies have revealed that tPA promotes the aggregation of annexin A2 with cluster of differentiation molecule (CD)11b, an integrin receptor, leading to activation of the downstream integrin-linked kinase (ILK) and eventually the NF-κB pathway [72]. In the classic UUO model of CKD, obstruction-induced NF-κB activation, as indicated by in vivo phosphorylation of p65 and expression of IP-10, is greatly decreased in tPA knockout mice in comparison with their wild-type littermate controls [72]. These findings have identified a novel signaling mechanism of NF-κB activation by tPA in promoting macrophages accumulation and renal inflammation (Figure 3A).

#### 4.3.1. tPA and NF-κB Promote Macrophage Motility in Obstructive Kidney Diseases

Previous work has shown that tPA is upregulated during the onset of macrophage and neutrophil infiltration in various injury models, indicating a role of tPA in this process [31,72,73]. Our recent work has shown that myeloid cell-derived tPA modulates macrophage motility through its protease-independent cytokine function [74]. Further mechanistic studies have revealed that tPA activates CD11b integrin signaling by phosphorylating its downstream focal adhesion kinase (FAK) and sequentially activating Ras-related C3 botulinum toxin substrate 1 (Rac1) [74]. FAK, an integrin signaling kinase, and Rac1, a Rho GTPase, are known to promote cell motility and spreading [75,76]. Intriguingly, NF-κB signaling is indispensable to tPA-induced macrophage motility, because NF-κB inhibition attenuates the effect of tPA in macrophages. This work identified a novel role for tPA as a macrophage motility facilitator through activating the FAK/Rac-1/NF-κB pathway (Figure 3B).

#### 4.3.2. tPA and NF-κB Modulate Renal Macrophage Polarization

Macrophages are cells with variably mixed populations, such as liver Kupffer cells and brain microglial cells, which carry out specific functions in the local microenvironment [77]. In response to various physiological or pathological cues, macrophages display an extended life span and acquire different functional phenotypes through a process called macrophage polarization that are generally categorized into two broad but distinct subsets as either classically activated (M1) or alternatively activated (M2). In general, M1 macrophages have high motility and promote inflammation and damage through a combination of transcription factors such as NF-κB, whereas M2 macrophages help to resolve inflammation and promote tissue remodeling [78,79]. M1 macrophage accumulation has been documented during early stages of kidney injury, while M2 macrophages are more prevalent during later stages of injury as there is effort to resolve inflammation and promote repair [80,81]. While the mechanism underlying macrophage polarization remains not completely understood, our previous work has demonstrated that tPA preferably promotes M1 macrophage survival leading to profound inflammation in an obstruction-induced CKD model, suggesting a potential role of tPA in macrophage polarization [69]. In addition, the fact that the tPA/NF-κB pathway has been shown to promote macrophage motility, a typical characteristic of M1 macrophages, further supports their role in such process [74]. To this end, we have investigated the subsets of macrophages during obstruction-induced CKD progression and have discovered that tPA promotes M1 macrophage accumulation in the fibrotic kidneys with concurrent induction of proinflammatory chemokines, such as inducible nitric oxide synthase (iNOS), TNF-α, and interleukin (IL)-1β [82]. Furthermore, tPA has been shown, in vitro, to induced macrophage phenotypic change of M2 to M1 [82]. In exploring the mechanism(s) behind these findings, we have revealed that annexin A2 and NF-κB mediates tPA-induced macrophage polarization from M2 to M1 [82]. This finding has defined another previously unknown function for tPA and NF-κB in macrophage differentiation during CKD (Figure 3C).

## 5. tPA and NF-κB in Other Diseases

### 5.1. tPA and NF-κB in Cerebral Ischemic Stroke

In terms of CNS diseases, tPA is widely recognized as the only FDA approved treatment for ischemic stroke since the mid 1990’s [27]. The success of tPA in treating ischemic stroke is primarily due to its ability as a protease to intervene and breakdown blood clots. However, treating with tPA can cause a side effect in 2%–7% of patients resulting in an intracranial hemorrhage, the leading cause of death in ischemic stroke patients treated with tPA [83]. It is presumable that excessive fibrinolysis induced by tPA, as well as impaired vascular endothelial integrity, contributes to intracranial hemorrhage. Fibrinolytic products not only consume coagulation proteins and platelets, but also interfere clot formation through inhibiting the crosslink of fibrin. However, the exact mechanisms by which tPA can induce cerebral hemorrhaging and other side-effects are still not entirely understood [84]. Understanding how tPA causes these serious, negative side-effects is of great importance given that it is the only approved FDA drug. Therefore, there has been a large body of research into the role of tPA in the CNS. In brief, existing studies have found that tPA is involved in many CNS processes including neuronal plasticity and neuron degeneration/death [85,86]. tPA, in conjunction with its associated receptor LRP-1, has also been shown to induce damage to the blood-brain barrier by increasing its permeability [87]. Of interest, key immune cells which are incorporated during cerebral ischemia, including microglia and neutrophils, have also been shown to be activated by tPA [32,40,88].

The pathogenesis of ischemic stroke begins with a complex orchestration of inflammatory responses. Thus, the involvement of NF-κB has been progressively explored. In cerebral ischemia, the NF-κB pathway is activated, which is generally considered to further promote neuronal cell death [89]. However, recent work from Pizzi group indicates a dual role of NF-κB members as either cell death- or cell survival-promoting factors [90]. It has been shown that c-Rel-containing dimers, such as p50/c-Rel or RelA/c-Rel, promote neuroprotection through induction of anti-apoptotic gene expression, whereas, p50/RelA dimer promotes neurotoxicity through the transcription of pro-apoptotic genes [91]. Intriguingly, post-transcriptional modification of RelA, such as acetylation of K310, has been shown to be critical to the cellular decision to fight or capitulate to ischemic brain injury [92,93]. 

Recent findings suggest that tPA modulates the NF-κB pathway and inflammation during ischemic brain injury. Zhang et al., in 2007, first linked tPA and NF-κB when they found that in an animal model of middle cerebral artery occlusion (MCAO), ischemia-induced NF-κB activation was significantly alleviated in tPA-deficient mice [32]. This effect was independent of plasminogen, indicating that tPA likely acted as a cytokine to influence NF-κB activation [32]. LRP-1 was responsible for the effect of tPA in this context, leading to NF-κB-dependent expression of iNOS [40]. This connection is important because previous studies on ischemic brain injury found that iNOS expression is increased after injury and that in mice lacking iNOS there was diminished neurological damage, implying that iNOS expression is an important determinant factor during ischemic brain injury [94].

Recent studies have explored the potential of using an isoflurane pretreatment before tPA treatment to combat the negative side effects [95]. This is a very promising combination because isoflurane has previously been used in the treatment of ischemic reperfusion injury of other organs [96]. Using oxygen/glucose deprivation and reperfusion (OGD/R) in brain endothelial cells to mimic ischemic stroke, Cheon et al. in 2017 found that pretreatment with isoflurane protects these cells from death. Mechanistically, they demonstrate that isoflurane pretreatment suppresses tPA-induced activation of LRP-1 and NF-κB signaling in OGD/R cells [95]. This work not only validates the pathogenic role of tPA/LRP-1/NF-κB in ischemic stroke, but also points to a potential new therapeutic strategy. Of note, desflurane or sevoflurane has replaced isoflurane in clinical settings, however, their therapeutic efficacy remains to be validated.

### 5.2. tPA and NF-κB in Cardiovascular Diseases

Cardiovascular disease (CVD) is the leading cause of mortality worldwide. Identifying CVD risk factors is an essential part for the prevention, early diagnosis, and timely intervention to avoid further medical complications. An elevated tPA level has been shown to be associated with atherosclerosis and considered as a potential indicator for myocardial infarction (heart attack) [97,98]. Lowe and colleagues have performed a meta-analysis study showing a significant association between CVD and tPA level, however, whether tPA is an independent risk factor for CVD remains to be elaborated [99]. A recent analysis has identified both tPA and plasminogen activator inhibitor-1 (PAI-1), a fibrinolytic inhibitor that negatively regulates tPA activity, as important risk factors for CVD [100]. NF-κB signaling has also been implicated in the pathogenesis of atherosclerosis, because inflammation plays a critical role in the process [101]. Myocardial infarction and reperfusion injury induces proinflammatory cytokines, such as TNFα, to activate NF-κB signaling leading to increased inflammation [102]. Although many studies have observed the concurrent induction of tPA and NF-κB in CVD, their direct interaction in mediating CVD remains largely unknown. However, a study focused on inflammatory cytokines in vascular endothelial cells has demonstrated a correlation between NF-κB signaling and tPA mRNA expression, indicating that NF-κB may play a role in regulating tPA expression [103]. This provides an interesting new context for tPA/NF-κB signaling and implies that their interaction may be cell context dependent.

## 6. Conclusions

It is clear that tPA and NF-κB independently and cooperatively modulate many diverse cellular processes and play critical roles in the pathogenesis of human kidney, CNS, and cardiovascular diseases. Future studies are warranted to investigate the signaling details of the interactions between tPA and NF-κB, as these studies will certainly point to the development of promising and effective therapeutic strategies.

## Abbreviations

The following abbreviations are used in this manuscript:

AKI	acute kidney injury
BAFFR	B cell activating factor receptor
CCL21	chemokine (C-C motif) ligand 21
CD11b	cluster of differentiation molecule 11b
CKD	chronic kidney disease
CNS	central nervous system
CVD	cardiovascular disease
ECM	extracellular matrix
EGF	Epidermal growth factor
ERK	extracellular signal-regulated kinase
ERSD	end-stage renal disease
FAK	focal adhesion kinase
FDA	U.S. Food and Drug Administration
Fn14	fibroblast growth factor-inducible factor 14
HGF	hepatocyte growth factor
IBD	inflammatory bowel disease
IκB	inhibitory κB
IKK	inhibitory κB kinase
IL-1 β	interleukin 1β
ILK	integrin-linked kinase
iNOS	inducible nitric oxide synthase
IP-10	interferon-γ-inducible protein 10
I/R	ischemia/reperfusion
LRP-1	low density lipoprotein receptor-related protein 1
LTβR	lymphotoxin-β receptor
MCAO	middle cerebral artery occlusion
MCP-1	monocyte chemoattractant protein 1
MIP-1	macrophage inflammatory protein 1
MMP	matrix metalloproteinase
NEMO	NF-κB essential modulator
NF-κB	nuclear factor-κB
NIK	nuclear factor -κB inducing kinase
OGD/R	oxygen/glucose deprivation and reperfusion
p90RSK	the 90kDa ribosomal s6 kinase
PAI-1	plasminogen activator inhibitor-1
PAMPs	pathogen-associated molecular patterns
PRRs	pattern-recognition receptors
RHD	Rel homology domain
Rac1	Ras-related C3 botulinum toxin substrate 1
ROS	reactive oxygen species
TAD	transactivation domain
TCR	T-cell receptor
TLR	toll-like receptor
TNF	tumor necrosis factor
TNFR	tumor necrosis factor receptors
tPA	tissue plasminogen activator
TWEAK	TNF-like weak inducer of apoptosis
uPA	urokinase plasminogen activator
UUO	unilateral ureteral obstruction
UV	ultraviolet
